# Validity of the Established Method of Quantifying Home Advantage in Soccer

**DOI:** 10.1515/hukin-2015-0001

**Published:** 2015-04-07

**Authors:** Richard Pollard, Miguel-Ángel Gómez

**Affiliations:** 1 Department of Statistics, California Polytechnic State University, San Luis Obispo, USA.; 2 Faculty of Physical Activity and Sport Sciences, Polytechnic University of Madrid, Spain.

A recent letter published in the Journal of Human Kinetics (Saavedra et al., 2014) falsely claims that inconsistencies occur in a universally accepted method of calculating home advantage when three points are awarded for a win. They go on to claim that an alternative method that they advocate is superior. The good news is that the authors have finally spelt out their methodology for calculating home advantage, so that readers can now see for themselves why it is not, in fact, a quantification of home advantage at all.

The reason why their method makes no sense is that performance at home has to be assessed against performance away from home for a measure of home advantage to be valid. Suppose team A gains 40 points at home and 40 points away from home, and team B scores 40 points at home and 10 away from home. According to Saavedra et al. (2014) these two teams have the same home advantage because they gained the same number of points at home. However, it should be obvious that team A actually has no home advantage at all, since it does equally well away as it does at home, while team B has a big home advantage since it gains four times as many points at home than it does away. The method of Pollard (1986) quantifies home advantage as the number of points won at home expressed as a percentage of all points gained at home and away. Thus, team A would have a value of 50% and team B 80%.

The two so-called ‘axioms’ that the authors state at the start of their letter make it clear that they have no concept of what constitutes home advantage as opposed to home performance which is what they are quantifying. Both their Tables 1 and 2 need an additional column headed ‘away points’ before home advantage can be calculated. This is what was correctly done by both Pollard (1986) and Sánchez et al. (2009) whose figures in these tables are valid measures of home advantage, in contrast to the Saavedra values which are not.

Saavedra et al. (2014) go on to criticize the paper by Pollard (1986) on the grounds that draws in cricket and US football were not considered when calculating home advantage. This paper was written almost 30 years ago and should be set in context. It was the first paper in which an attempt was made to compare home advantage in different sports in different leagues in different countries. The main focus of the paper was soccer in England and it introduced the method of calculating home advantage when three points were awarded for a win and one to each team for a draw. This method, to which the authors of the letter now object, has been used by dozens of researchers in dozens of peer reviewed international journals during the last three decades and is one of several perfectly valid measures of quantifying home advantage. Moreover, Pollard (1986) has been cited 86 times in the Web of Science, and 283 times according to Google scholar citations and the method has never been questioned. With regard to draws, if readers were to revisit the paper by Pollard (1986), they would see that in US football during the three years under study, only three games ended in a draw, well less than 1% of all games played. The inclusion of these three drawn games, or not, in the calculation of home advantage would make virtually no difference to the value of 55.0% given. With regard to cricket in England, the County Championship played an unbalanced schedule of games during the three years studied. Furthermore, there was a complicated system of points allocation in use, so that a winning team gained between 12 and 24 points, a losing team between 0 and 8 points while a draw was also worth between 0 and 8 points, but not necessarily the same number for each team. Rather than attempt to incorporate all this into a quantification of home advantage, it was decided to simply use wins and losses as a best estimate.

In their earlier letter to the Journal of Human Kinetics, Gómez and Pollard (2014) pointed out that the claim by Saavedra et al. (2013) that better quality teams have higher home advantage must be false. Since the authors have not responded to this we assume they now accept that they were in error. If further evidence were needed on this point, a recent paper by Allen and Jones (2014) investigated this relationship for soccer in England and concluded that ‘teams finishing towards the lower end of the league table show a greater home advantage than teams finishing towards the higher end of the league table’. In other words, better quality teams have lower home advantage not higher. It should also be noted that Allen and Jones (2014) used the method introduced by Pollard (1986) to quantify home advantage, the exact same method which Saavedra et al. (2014) now wrongly claim has ‘inconsistencies’.

## References

[b1-jhk-45-07] Allen MS, Jones MV (2014). The home advantage over the first 20 seasons of the English Premier League: Effects of shirt colour, team ability and time trends. Int J Sport Exer Psychol.

[b2-jhk-45-07] Gómez MA, Pollard R (2014). Calculating the Home Advantage in Soccer Leagues. J Hum Kinet.

[b3-jhk-45-07] Pollard R (1986). Home advantage in soccer: a retrospective analysis. J Sports Sci.

[b4-jhk-45-07] Saavedra M, Gutiérrez O, Fernández JJ (2014). Inconsistencies of the evaluation of home advantage in sports competition under the three points per victory system. J Hum Kinet.

[b5-jhk-45-07] Saavedra M, Gutiérrez O, Sa Marquez P, Torres G, Fernández JJ (2013). Calculating home advantage in the first decade of the 21th century UEFA soccer leagues. J Hum Kinet.

[b6-jhk-45-07] Sánchez PA, García-Calvo T, Leo FM, Pollard R, Gómez M (2009). An analysis of home advantage in the top two Spanish professional football leagues. Percept Motor Skill.

